# Ending discrimination in healthcare

**DOI:** 10.1002/jia2.25471

**Published:** 2020-02-27

**Authors:** Joseph J Amon

**Affiliations:** ^1^ Office of Global Health Dornsife School of Public Health Drexel University Philadelphia PA USA

**Keywords:** human rights, health systems, key and vulnerable populations, law and policy, stigma, discrimination

In mid‐January, in a sparsely populated corner of northern Ghana, I met a 13‐year‐old girl named Hannah. I asked her about her responsibilities at home and her studies and how she imagined her future. Her response was quick. She said she would like to become a nurse. When I asked why, her answer was as prompt. She explained that a few months previously she had been sick and taken to a health clinic. The nurses there, she said, did not have a good attitude towards patients. She wanted to change that.

There are many reasons why the patient care Hannah experienced might have been less than optimal. While Ghana has invested significantly in increasing the number of nurses and midwives and exceeds the WHO's recommended nurse to population ratio, many challenges to ensuring quality care remain, including the training and mentoring of newly trained nurses 1. Another challenge is stigma and discrimination.

Stigma and discrimination may be due to multiple factors, but centres on the identification of an “other” and their devaluation. Stigma may be based on expectations of roles in society (e.g. racism), cultural norms (e.g. homophobia) and/or fears of contagion (avoidance of infectious diseases). In the case of HIV, stigma and discrimination may have multifactorial causes and expressions.

Stigma and discrimination have been much discussed in the HIV response, as well in public health interventions seeking to expand access to sexual and reproductive health and mental health services. Nonetheless, they remain a persistent obstacle to achieving the goal of universal health coverage and “leaving no one behind”. People living with HIV experience a range of stigmatizing experiences and discrimination within society, from social isolation to violence to denial of housing, employment and healthcare. They may also face police harassment or arrest in contexts where HIV transmission or specific behaviours are criminalized, and often confront intersecting stigma and discrimination due to other health conditions or identities, including gender, disability, race/ethnicity and sexuality. Recognizing this, in 2014 the United Nations selected 1 March as Zero Discrimination Day.

Admittedly, while governments worldwide have an obligation to eliminate all forms of discrimination stemming from their ratification of human rights treaties as well as constitutional protections and laws, achieving zero discrimination is a tough task. More narrowly, increasing focus has been put on ending discrimination in health settings.

The 2016 United Nations Political Declaration on Ending AIDS called on member nations to commit to eliminating stigma and discrimination in healthcare settings 2. Following this pledge, the Global Partnership for Action to Eliminate All Forms of HIV‐Related Stigma and Discrimination was formed with the participation of the United Nations Development Programme (UNDP), the United Nations Entity for Gender Equality and the Empowerment of Women (UN Women), the Global Network of People Living with HIV (GNP+), the Joint UN Programme on HIV/AIDS (UNAIDS) and non‐governmental partners 3. The Global Fund's Breaking Down Barriers Initiative has also targeted discrimination, funding interventions on stigma and discrimination reduction, training for healthcare providers on human rights and medical ethics, sensitization of law‐makers and law enforcement agents, as well as legal literacy, legal services, and law reform 4, interventions identified by UNAIDS as essential for every national AIDS response 5.

A recent review found evidence of the impact of these types of human rights programmes (singly and combined) on HIV‐related outcomes for people living with HIV and key and vulnerable populations most at risk of HIV, ranging from decreased HIV risk behaviours to increased HIV testing to reduced incidence 6. The review examined research published between 2003 and 2015, but evidence of the positive impact of similar interventions both prior to and after these dates have also been published; for example, focusing on the training of health workers to reduce stigma 7, 8, 9, 10, 11, 12 and programmes promoting legal literacy and advocacy 13, 14. Advocacy targeting discriminatory laws, policies and practices have also been shown to be effective to removing barriers to HIV services 15, while evidence of the effectiveness of sensitizing law enforcement is increasing 16.

Yet, adequately funded human rights programmes addressing discrimination operating at national scale are rare. More often, “stigma and discrimination” programmes are small or *ad hoc* and emphasize stigma 17, 18, 19, 20 but ignore discriminatory laws, policies and practices. They rely on messaging that calls on everyone to act together to end stigma, while ignoring mechanisms, such as the judiciary, that can identify and hold responsible those who discriminate against others. Making everyone responsible usually means that no one is accountable.

To truly achieve zero discrimination in health settings, governments and health settings need to “own” the issue and commit to action. Accountability measures need to be created so that patients, such as Hannah, have a place to turn to complain about being denied care or treated poorly 21. Integrating paralegals in health facilities, creating ombudsman's offices, posting – and respecting – a patient's bill of rights in health facilities, combined with independent monitoring and civil society advocates, would begin to make Zero Discrimination Day real. Achieving an “end to AIDS”, and all health‐related sustainable development goals, requires a commitment to available, accessible, acceptable and quality care for all who need it.

We know what discrimination in health settings looks like: delays in treatment, disrespectful care, verbal and physical abuse and outright denial of care. We know that programmes that train healthcare providers, that promote legal literacy and provide legal services and that reform discriminatory laws and policies and ensure legal protections are effective. We need government leaders willing to take a stand. Or 1 March will be just another day falling between International Mother Language Day and World Wildlife Day.

## Competing interests

The author has no competing interest.

## Author's contribution

JJA conceptualized, wrote and approved the article.

## Author's information

Joseph J. Amon is the Director of Global Health and Director of the Jonathan Mann Global Health and Human Rights Initiative at the Dornsife School of Public Health at Drexel University. He serves as co‐chair of the UNAIDS Human Rights Reference Group on HIV and Human Rights and is a member of the Working Group on Monitoring and Evaluating Programmes to Remove Human Rights Barriers to HIV, TB and Malaria Services of the Global Fund.

## References

[1] Asamani JA , Amertil NP , Ismaila H , Francis AA , Chebere MM , Nabyonga‐Orem J . Nurses and midwives demographic shift in Ghana—the policy implications of a looming crisis. Hum Resour Health. 2019;17(1):32. 31118024 10.1186/s12960-019-0377-1 PMC6530167

[2] Joint United Nations Program on HIV/AIDS . Agenda for zero discrimination in health‐care settings. 2017 [cited 2020 Jan 29]. Available from: https://www.unaids.org/sites/default/files/media_asset/2017ZeroDiscriminationHealthCare.pdf

[3] UNAIDS . Global Partnership for eliminating all forms of HIV‐related stigma and discrimination. no date. [cited 2020 Jan 29]. Available from: https://www.unaids.org/sites/default/files/media_asset/global-partnership-hiv-stigma-discrimination_en.pdf

[4] Global Fund . Global Fund, Technical Brief: HIV, Human Rights and Gender Equality (October 2019). [cited 2020 Jan 29]. Available from: https://www.theglobalfund.org/media/1213/humanrights_2016-removingbarrierspart2_qa_en.pdf; https://www.theglobalfund.org/media/6348/core_hivhumanrightsgenderequality_technicalbrief_en.pdf

[5] UNAIDS . Key programmes to reduce stigma and discrimination and increase access to justice in national HIV responses. Guidance note. 2012 [cited 2020 Jan 29]. Available from: https://www.unaids.org/sites/default/files/media_asset/Key_Human_Rights_Programmes_en_May2012_0.pdf

[6] Stangl AL , Singh D , Windle M , Sievwright K , Footer K , Iovita A , et al. A systematic review of selected human rights programs to improve HIV‐related outcomes from 2003 to 2015: what do we know? BMC Infect Dis. 2019;19(1):209. 30832599 10.1186/s12879-019-3692-1 PMC6399958

[7] Ezedinachi E . The impact of an intervention to change health workers' HIV/AIDS attitudes and knowledge in Nigeria a controlled trial. Public Health. 2002;116:106–12. 11961679 10.1038/sj.ph.1900834

[8] Williams AB , Wang H , Burgess J , Wu C , Gong Y . Effectiveness of an HIV/AIDS educational programme for Chinese nurses. J Adv Nurs. 2006;53:710–20. 16553679 10.1111/j.1365-2648.2006.03777.x

[9] Oanh KTH , Ashburn K , Pulerwitz J , Ogden J , Nyblade L . Improving hospital‐ based quality of care in Vietnam by reducing HIV‐related stigma and discrimination, a Horizons final report. Washington DC: Population Council; 2008.

[10] Lohiniva AL , Benkirane M , Numair T , Mahdy A , Saleh H , Zahran A , et al. HIV stigma intervention in a low‐HIV prevalence setting: a pilot study in an Egyptian healthcare facility. AIDS Care. 2016;28(5):644–52. 10.1080/09540121.2015.1124974 26717980

[11] Batey DS , Whitfield S , Mulla M , Stringer KL , Durojaiye M , McCormick L , et al. Adaptation and implementation of an intervention to reduce HIV‐related stigma among healthcare workers in the United States: piloting of the FRESH Workshop. AIDS Patient Care STDS. 2016;30(11):519–27. 10.1089/apc.2016.0223 27849373 PMC5116658

[12] Geibel S , Hossain SMI , Pulerwitz J , Sultana N , Hossain T , Roy S , et al. Stigma reduction training improves healthcare provider attitudes toward, and experiences of, young marginalized people in Bangladesh. J Adolesc Health. 2017;60(2):S35–S44. 10.1016/j.jadohealth.2016.09.026 28109339

[13] Feinglass E , Gomes N , Maru V . Transforming policy into justice: the role of health advocates in Mozambique. Health Hum Rights. 2016;18(2):233. 28559689 PMC5394992

[14] Goodwin L , Maru V . What do we know about legal empowerment? Mapping the evidence. Hague J Rule of Law. 2017;9(1):157–94.

[15] Amon JJ , Wurth M , McLemore M . Evaluating human rights advocacy on criminal justice and sex work. Health Hum Rights. 2015;17(1):91. 26204588

[16] Crofts N , Patterson D . Police must join the fast track to end AIDS by 2030. J Int AIDS Soc. 2016;19:21153. 27435719 10.7448/IAS.19.4.21153 PMC4951534

[17] Nyblade L , Stangl A , Weiss E , Ashburn K . Combating HIV stigma in health care settings: what works? J Int AIDS Soc. 2009;12(1):15 10.1186/1758-2652-12-15 19660113 PMC2731724

[18] Ekstrand ML , Ramakrishna J , Bharat S , Heylen E . Prevalence and drivers of HIV stigma among health providers in urban India: implications for interventions. J Int AIDS Soc. 2013;16:18717 10.7448/IAS.16.3.18717 24242265 PMC3833193

[19] Li L , Liang LJ , Lin C , Wu Z . Addressing HIV stigma in protected medical settings. AIDS Care. 2015;27(12):1439–42. 10.1080/09540121.2015.1114990 26608559 PMC4689649

[20] Varas‐Díaz N , Neilands TB , Cintrón‐Bou F , Marzán‐Rodríguez M , Santos‐Figueroa A , Santiago‐Negrón S , et al. Testing the efficacy of an HIV stigma reduction intervention with medical students in Puerto Rico: the SPACES project. J Int AIDS Soc. 2013;16:18670 10.7448/IAS.16.3.18670 24242260 PMC3833102

[21] Hunt P . SDG series: SDGs and the importance of formal independent review: an opportunity for health to lead the way. Health Hum Rights J. 2015.

